# The role of design patterns in the development and legal assessment of lawful technologies

**DOI:** 10.1007/s12525-022-00597-1

**Published:** 2022-10-20

**Authors:** Ernestine Dickhaut, Mahei Manhai Li, Andreas Janson, Jan Marco Leimeister

**Affiliations:** 1 Information Systems, ITeG, University of Kassel, Kassel, Germany; 2 Institute of Information Management, University of St. Gallen, Müller‑Friedberg‑Strasse 8, 9000 St. Gallen, Switzerland

**Keywords:** Design pattern, Smart personal assistants, Lawful system development, Digital services, Law simulation study, D47, D8, K1

## Abstract

Novel technologies such as smart personal assistants integrate digital services into everyday life. These services use personal data to offer personalized services. While they are subject to special data protection regulations at the time of development, there are few guidelines describing the transition from legal requirements to implementation. To reduce risks, services depend on external legal assessments. With developers and legal experts often missing either legal or technical knowledge, the challenge lies in bridging this gap. We observe that design patterns support both developers and legal experts, and we present an approach in which design patterns are leveraged to provide twofold value for both developers and legal experts when dealing with novel technologies. We conducted a revelatory case study for smart personal assistants and scaffolded the case interpretation through cognitive fit theory. On the basis of the findings, we develop a theoretical model to explain and predict the twofold value of design patterns to develop and assess lawful technologies.

## Introduction

Digital services are an essential part of today’s connected world. Novel technologies such as smart personal assistants (SPAs) make digital services permanent everyday companions (Janssen et al., 2020; Skjuve et al., 2021). SPAs support the user in various ways; voice-based assistants such as Amazon’s Alexa use smart light bulbs or sockets to turn a home into a smart home, Google Assistant or Apple’s Siri run on the cell phone and accompany the user everywhere, text-based chatbots automate basic customer interactions, and the virtual fitness assistant on a smartwatch accompanies the user on their journey to becoming more athletic (Knote et al., 2021). However, these typically platform-based digital services process a large amount of data to offer their personalized services (Gimpel et al., 2018), including much personal data, which requires special protection (Baruh et al., 2017).

Higher legal standards, such as the General Data Protection Regulation (GDPR) in the European area, regulate the processing, storing, and managing of personal data (GDPR, 2018) but influence information systems (IS) developments all over the world (Peukert et al., 2022). Recent privacy scandals and high penalties show the importance for companies and providers of digital services to consider these legal rules (Baruh et al., 2017). Thus, legal requirements are increasingly gaining influence on the development of novel technologies and ultimately decide the market approval of these technologies (Hildebrandt & Tielemans, 2013; Human & Kazzazi, 2021). The GDPR requirements form a framework written in legal jargon that must be interpreted for each specific application. Oftentimes, companies hire external data protection officers who are educated lawyers to prevent GDPR violations and the associated penalties. However, these lawyers only give limited guidance for the implementation in the technology, so it is often difficult to analyze the legality in depth. This leads us to another challenge: apart from the actual development of a technology, the assessment of its lawfulness is a decisive step in determining whether a system is market-ready and sustainable through compliance with the law. Therefore, we see two points that are crucial for the development and approval of novel technologies such as SPAs: first, the consideration of legal requirements during development and, second, the legal assessment by legal experts (as an umbrella term for lawyers, judges, and other legal experts).

In this context, design patterns as proven solutions for recurring problems by making complex domain knowledge accessible and applicable for non-domain experts (Schoonderwoerd et al., 2022) could be a feasible way to improve the design and assessment of IT artifacts. The patterns could ensure the legality of norms such as the GDPR, and they could also contribute to sustainable, lawful IS. Up until now, knowledge related to the value of design patterns has mostly looked at supporting system development. This is an important gap, since both the development and the legal assessment play a decisive role in bringing novel digital services to market. Developers, as well as legal experts, can benefit from the use and further understanding of design patterns, practically and theoretically. By providing design patterns with legal and technical knowledge, i.e., legal design patterns that make legal knowledge accessible for developers, the added value of the patterns not only supports the developers but also supports legal experts in understanding the complex socio-technical systems, e.g., to argue about technical facts in court cases but also a priori when assessing newly developed IT artifacts. The goal of our paper is to analyze how design patterns provide a twofold value for developers and legal experts in their work dealing with novel technologies, such as SPAs. Further, we have the goal to abstract upon this analysis to develop an according theoretical model based on cognitive fit theory as a novel contribution to the field and to fill the important theory gap mentioned before. In consequence, we base our work on the following research question (RQ): What are the mechanisms that further the twofold value of legal design patterns from the development to the assessment of novel technologies?

To answer our research question, we conduct a revelatory case study and scaffold the case interpretation through cognitive fit theory. First, in the case study, developers use design patterns to develop an SPA as a learning assistant for higher education. Second, we provide the same design patterns as support for lawyers in court cases to investigate the application of the design patterns by legal experts. For this purpose, we use a law simulation study, which is a well-known evaluation method among law researchers for capturing the lawfulness of IT artifacts (Pordesch et al., 1999). The simulation study provides us with an evaluation strategy to evaluate aspects such as legality and data policy issues that have become important criteria for system development. The method is characterized by the fact that it allows the creation of realistic usage situations while real damage is prevented through the simulation of legal violations. We expect that the design patterns will provide solutions for recurring problems in the development and offer details for the technical implementation and corresponding explanations in the legal assessment. Thus, we contribute to theory by developing a theoretical model that demonstrates the interaction between novel technologies and existing legislation. The model maps the impact of design patterns on the development and legal assessment of technologies. For this, we use cognitive fit theory to investigate how to solve a missing cognitive fit (Shaft & Vessey, 2006) between internal and external representations by using design patterns as a bridge between law and technology. In addition, we contribute to practice by deriving insights into how design patterns support the development and negotiation of technologies in court cases and how they white-box the development of complex IT artifacts by making the procedure and the details of the development accessible to external parties.

## Related work and theoretical background

### Legal design challenges of smart personal assistants

As digital service interfaces, SPAs have permeated many people’s everyday lives. SPAs can support everyday life in many ways, such as on smartphones, in cars, in service encounters, in smart home environments, or as support for elderly or impaired people (Knote et al., 2021; Purington et al., 2017; Skjuve et al., 2021). With the GDPR, which came into force in 2018, developers of SPAs have had to pay more and more attention to legal requirements. Key aspects of SPAs relate, for example, to their usability and user experience, which we sum up with the overall term “service quality”, an important boundary condition from service design when developing novel service interfaces. Current SPAs that are widely available on the market pay much attention to service quality requirements, such as voice recognition, innovating SPA skills, and additional smart home devices. However, there is also growing skepticism and concern that these systems, for example, "listen" without being activated by a wake word (Foehr & Germelmann, 2020), thus showing that quality perceptions are difficult to achieve with these devices. More importantly, legal requirements for smart services such as SPAs are often only addressed to a minimum extent in order to be compliant with the minimal requirements (Hoffmann et al., 2015).

In this context, system developers often lack domain expertise to implement legal requirements for developing a lawful SPA (Aljeraisy et al., 2021). Higher legal standards with regards to the data protection of individuals such as the GDPR are increasing the pressure on developers of IT artifacts (Kühling & Martini, 2016). Furthermore, there is a lack of research on how to support developers in their design process of user assistance systems such as SPAs (Maedche et al., 2016). Both novelty and special features of SPA systems give rise to new legal design issues for which developers often lack support. In that sense, we have to further the understanding of how the infusion of legal design knowledge could work and what the boundary conditions are to make it work in practice.

### Legal design patterns as an approach for solving legal design challenges

**Fig. 1:**
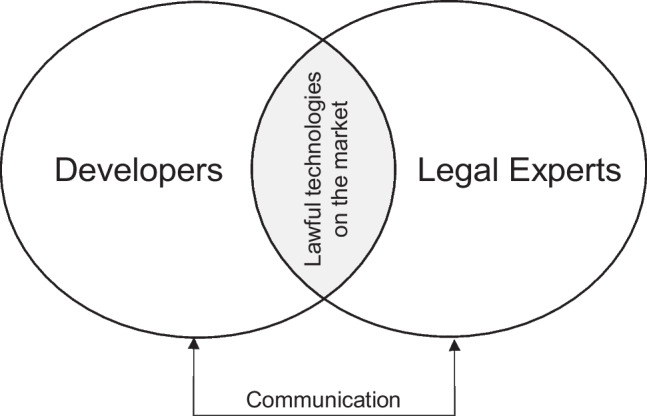
Challenges of bringing lawful technologies to market (based on Knackstedt et al. (2014))

When referring to the understanding and use of legal knowledge in any context, we often think of abstract legal texts that are difficult for nonexperts to understand.^1^ In particular, the legal requirements for data protection and data processing have drastically changed in recent years (Politou et al., 2018). However, legal norms, as part of social systems, are becoming increasingly important in user-centered system development (van der Sype & Maalej, 2014). In this context, lawfulness means that the legality of a system meets the minimum legal requirements to be approved for the market. With this intention, approaches should be followed that enable developers to extract legal knowledge for the development of lawful systems (Hafiz, 2006; Rossi et al., 2019; Yskout et al., 2015). In practice, legal aspects are often only taken into account at the end of development (Becker et al., 2014). The consequences of picking up such special requirements late in development processes are simple: requirements are usually only addressed to the extent of meeting the minimum amount of law requirements for the system to be brought to market. Thus, legal experts are often consulted in various development phases for the assessment of novel technologies. In legal assessments, legal experts have the necessary legal knowledge, but they often lack insights and the technical background of the system development of the technologies being assessed (see Fig. 1). Knackstedt et al. (2014) identify a lot of potential for misunderstandings and errors in the communication between legal experts and laymen, which can be caused by legal jargon.^2^

To overcome this issue on the development side, legal design knowledge should be prepared and made easily accessible to laymen as, for instance, service design knowledge is made accessible for the management discipline (Teixeira et al., 2017). For example, while SPAs have been on the market for many years, and during this time there have been many legal violations that have led to costly system revisions, it was only in 2021 that the European Data Protection Board (2021) provided an official guideline to provide guidance for developers concerning the design of these systems. On the legal side, challenges arise due to the overwhelming number of new technologies and obscure backgrounds of data processing and storage by, e.g., artificial intelligence approaches or machine learning. Legal experts lack interpretations of the law for new technologies, so they have to spend a lot of time and effort to familiarize themselves with the functionalities and backgrounds of the technologies.

There are various approaches to supporting lawful system development (see for an overview Table 1). For example, Hoffmann et al. (2015) developed legal requirement patterns to support the elicitation of legal aspects during requirement engineering. Other approaches rely on conceptual modeling to support lawful system development. Becker et al. (2014) suggest the integration of legal requirements through meta-designs to provide a structure and guidance to integrating a legal perspective into information systems, and Knackstedt et al. (2014) leverage conceptual modeling to bridge the knowledge gaps between lawyers and developers. Yet other approaches rely on tools, such as Bartolini et al. (2016), who apply natural language semantics techniques to process legal documents and standards to support legal experts in tracking legal requirements by assessing which requirements of the law have been met by the standard and which requirements still need to be implemented. Huth et al. (2020) more organically integrate legal aspects into system development by presenting an approach on how privacy aspects may be integrated into agile development practices, and Rossi et al. (2019) integrate legal aspects directly into design patterns. All these approaches share the goal of improving lawful system development by providing developers with support in implementing lawful requirements.

**Table 1:** Approaches to support lawful system development

Study	Approach
Huth et al. (2020)	Integrating privacy aspects in agile development
Knackstedt et al. (2014)	Using conceptual modelling to analyze lawful system development
Bartolini et al. (2016)	Framework using natural language semantics techniques to organize legal documents and standards
Hoffmann et al. (2015) ↓	Requirement patterns to support recurrent legal requirement engineering
This Study ↑	Design patterns that provide twofold value for both developers and legal experts when dealing with novel technologies
Rossi et al. (2019)	Legal design patterns for making contracts, disclosures and policies accessible

As Hoffmann et al. (2015) and Rossi et al. (2019) recognize, pattern-based approaches are interesting candidates for recurring legal design challenges, since it addresses specific important legal issues with established solutions while simultaneously ensuring that the developer retains enough freedom of choice during its implementation. Additionally, several researchers demonstrate that legal design patterns capture legal design knowledge and make legal issues more accessible to developers (Huth, 2017; Rossi et al., 2019). Design patterns originated in architecture as a means of solving recurring problems (Alexander, 1979) and have long been established in system development (Gamma, 1994; Wania, 2019) or other IS-related disciplines such as human–computer interaction (Dearden & Finlay, 2006). Thus, design patterns might differ in their representation, but the patterns’ core is the proven solution for recurring problems. The design pattern development process can be either inductive or deductive (Petter et al., 2010). Using a deductive approach, design patterns could present proven design solutions by concretizing European law to solve legal design issues (Hoffmann et al., 2015). Owing to the specification of law through fundamental rights and legislation, legal requirements stabilize over time and occur repeatedly in many development contexts in a similar form (Hoffmann et al., 2015). Thus, design patterns offer a promising solution to recurring legal requirement problems and may eliminate uncertainties affecting the implementation of legal requirements. Design patterns identify and structure design problems and provide an abstract solution so as to not restrict a developer’s design creativity.

While previous research depicts the challenge as a rather one-sided transformation of legal knowledge (from legal experts) into actionable design knowledge (to developers), we propose to analyze the holistic approach of legal design patterns that is easily accessible for both developers and legal experts. In other words, this paper aims to analyze a legal design-pattern-based approach that supports lawful system development holistically to develop a meaningful theory for understanding the two-fold value of legal design patterns. At the end of the day, a system needs to be developed so that it can hold up in court, not only to adhere to standards, which are often subject to change or lacking behind technological development. By using this dual value of design patterns in court cases and having it in mind in the development phase during its conceptualization, the resulting lawful design patterns need to rely on a common understanding of both disciplines. To better understand the revelatory case, we draw upon the theory of cognitive fit to scaffold our case analysis and conceptual development. Thus, we introduce in the next section cognitive fit as a theory grounding for the present paper.

### Cognitive fit as a scaffolding theory for understanding and solving legal design challenges

**Fig. 2:**
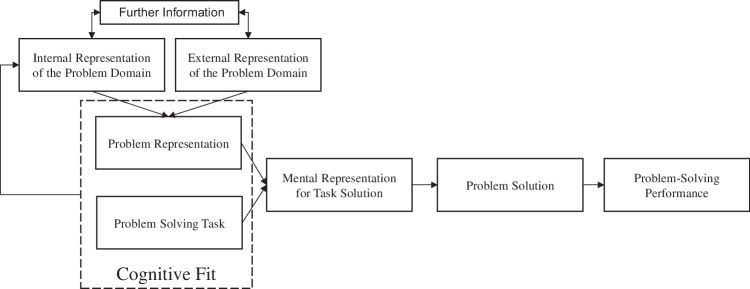
Cognitive fit theory and its underlying mechanism (based on Shaft and Vessey (2006))

Building upon the notion to better understand the issues of solving legal design challenges when being confronted with IS development, we draw upon cognitive fit theory as a scaffolding theoretical framework to help us in analyzing the revelatory case study and developing a conceptual model to capture the two-fold value of legal design patterns. First, cognitive fit theory was developed to understand how the fit between a task to be solved and its mental representation influences the skill to solve a problem (Vessey & Galletta, 1991). Accordingly, the performance of solving a problem depends on the representation of the problem and the task. If there is a mismatch between them both, the performance of problem-solving a specific task will suffer (Hong et al., 2004). Cognitive fit theory suggests that both the problem representation and the problem to resolve should correspond. A cognitive fit produces a consistent mental representation for problem-solving and, subsequently, leads to a faster and more accurate performance in decision-making (Agarwal et al., 1996). In recent decades, cognitive fit theory has been used to explain a wide range of problem-solving phenomena (Claes et al., 2015; Hong et al., 2004; Khatri et al., 2006; Shaft & Vessey, 2006) and is therefore a valuable theoretical basis when thinking about how problem-solving occurs during system development processes that especially involve multiple and sometimes conflicting perspectives (see Fig. 2).

Using Knackstedt et al.’s (2014) view on the communication between lawyers and laymen as a common ground between both parties would support bringing lawful technologies on the market (see Fig. 1). Thus, cognitive fit theory serves as a theoretical underpinning to understand the effects and boundary conditions of design patterns to solve legal design challenges. The design patterns act as common ground between developers and legal experts, as both sides understand the content of the other. Thus, a cognitive fit is achieved. By providing the developer with examples of solutions to legal problems, the developer can successfully implement legal aspects, or to put it in cognitive fit language: the mental representation leads to a faster problem-solving experience. Design patterns may provide legal experts with insights into the development, which are even linked with legal requirements, the problem representation and the problem to resolve correspond.

## Methodology

### Research design and case selection

For investigating our research question, we use a revelatory case study approach that aims to investigate the use of design patterns in two application contexts, namely developing lawful technologies and the legal assessment of technologies. First, we conducted a development project that developed a lawful SPA, more precisely a voice-based intelligent learning assistant for a university course. Thus, the SPA had to comply with the legal requirements of the university and was therefore subject to strict data protection regulations, such as the GDPR. For this purpose, the developers used a legal design pattern repository consisting of twelve legal design patterns that were developed in an interdisciplinary research team consisting of information systems, legal and computer science scholars on a deductive basis (Hoffmann et al., 2015) considering legal and service experience requirements (Dickhaut et al., 2020). To ensure the utility of the legal design patterns, the patterns were subject to multiple evaluation episodes including a proof of value and proof of concept evaluation, including also an experimental evaluation with IS designers.

In the second part of the case study, we accompany a law simulation study that evaluates the legality of the developed SPA in four simulated court cases. Simulation studies are a well-known evaluation method among law researchers for capturing the legality of IT artifacts (Pordesch et al., 1999). The study was carried out in court cases according to European law to meet the strict legal requirements such as the GDPR. In the simulation study, court cases with practicing lawyers and judges and a legal dispute were simulated to clarify the state of the facts.

Our case offers the opportunity to observe and analyze a phenomenon previously inaccessible to social science inquiry (Yin, 2018). According to the five components of case studies from Yin (2018), a research method is especially useful when the researcher has little or no control over behavioral events, the focus of the study is a contemporary phenomenon, and logic links the data to the propositions and criteria for interpreting the findings. This was true in our case: we had no influence on behavioral events, which were carried out independently of each other, as we did not interfere in the interactions between the developers, lawyers, and judges in the court cases; the development of lawful systems was and is still becoming increasingly important, particularly in Europe, where innovations in the GDPR have created stricter legal requirements for technologies; and the linking of our data to the proposition was done by pattern matching according to Yin (2018), and we used this strategy to identify, address, investigate, and (if appropriate) reject rival explanations to our findings.

### Data collection and analysis

According to our research design, our data collection can be divided into two independent phases: (1) the development of the SPA and (2) the use of the SPA by real users, resulting in four court cases to assess the legality of the SPA in court. Overall, we were able to accompany the entire development of the learning assistant, the use of the learning assistant by students in a university course, and the resulting simulated legal disputes arising from user complaints, which had to be clarified in court. This setting allowed us to gain comprehensive insights into the development and the legal assessment of the SPA, which faces particularly significant legal challenges. To pursue our research question, we triangulate our findings and use insights from different data sources: (1) we scaffold our case analysis interpretation with cognitive fit theory to gain an overview and identify relevant concepts related to problem-solving performance in our case, (2) we utilize the documentation of the development, (3) we analyze interviews with the involved developers, (4) we gather feedback from the users of the SPA, (5) we analyze four written pretrial proceedings and (6) the recordings of four court cases followed by (7) a group discussion with the involved lawyers and judges, and (8) interview the two involved judges. Table 2 summarizes the legal case and highlights the rich data sources used to enable data triangulation to support our findings and increase the validity of our insights. This data collection in the end supported us to develop a theoretical model for understanding the legal design patterns under IS development and legal considerations (see next section).

**Table 2:** Data overview of the revelatory case study

	Data Collection	Data	Participants	Scope/length
Inscribing Law		Documentation	Developer Team	-
		Notes	Developer Team	9 pages
	Development	Interview 1	Developer 1	37 min
		Interview 2	Developer 2	42 min
		Interview 3	Developer 3	30 min
	SPA Use	Questionnaires	SPA User	19 pages
Extracting Law		Filing of action of the case “disclosure of personal data”	Exchange of pleadings	7 pages
		Filing of action of the case “preventing the use of an AI-based assistant”	Exchange of pleadings	4 pages
	Pretrial	Filing of action of the case “storage period of personal data”	Exchange of pleadings	7 pages
		Filing of action of the case “right to data deletion”	Exchange of pleadings	6 pages
		Statement of defense of the case “disclosure of personal data”	Written hearing	5 pages
	Written hearing	Statement of defense of the case “preventing the use of an AI-based assistant”	Written hearing	5 pages
		Judgment of the case “disclosure of personal data”	Written hearing	2 pages
		Judgment of the case “preventing the use of an AI-based assistant”	Written hearing	3 pages
		Video recording of case “storage period of personal data”	Oral hearing	58 min
		Video recording of case “right to data deletion”	Oral hearing	46 min
	Oral hearing	Statement of defense of the case “storage period of personal data”	Oral hearing	5 pages
		Statement of defense of the case “right to data deletion”	Oral hearing	4 pages
		E-learning charter of the university	Oral hearing	2 pages
		Transcript of the case “storage period of personal data”	Oral hearing	10 pages
		Transcript of the case “right to data deletion”	Oral hearing	8 pages
	Interviews	6 Interview transcripts	Legal experts	5 pages

The development took place in the period from November 2019 to January 2020, and the development team (N = 3) consisted of two experienced developers with two and three years of development experience and one developer with little programming experience. During the development, we accompanied weekly meetings of the development team and received the documentation and other material accrued during the development, such as notes. After the completion of the SPA, we conducted semi-structured interviews to get extra insights into the development process and the design pattern use. We devised our interview guide in accordance with our overall goal and used questions to unravel the legal design challenges during the development and how the developers handled them. All semi-structured interviews were recorded and transcribed for coding purposes.

After the development, students from a university course used the developed SPA for exam preparation. Students could voluntarily participate in exam preparation and review the semester’s content with the assistance of the SPA. At the beginning, students created a profile on the SPA, which allowed for adaptive adjustments to their learning progress. The content was divided into several blocks in which voice-based playful content was repeated. The use of the SPA took place in February 2020. At the end, the participating students filled out a questionnaire that allows us to draw conclusions about the use, usability, and acceptance of the SPA.

Based on the SPA use, two legal experts created possible infringements against the GDPR and the university law i.e., the university-wide legal order for e-learning. Next, the legal experts prepared simulated court cases to judge the legality of the developed SPA. Six legal experts participated in our simulation study—two judges and four lawyers. The court cases took place in April 2020 and were conducted as online court cases due to the COVID-19 pandemic.

All participants (one female; five male) had completed the second state examination in law and had several years of professional experience. Each oral hearing lasted between 45 and 60 min. All participating lawyers received the design patterns that were used in the development and a note indicating that they were implemented in the learning assistant.

During the simulated court cases, we had the unique opportunity to look "behind the scenes" and question the involved persons about their experiences with the design patterns in their argumentation and judgment formation in the discourse. Just like in real court cases, written negotiations between the plaintiff, the defendant, and the judge were conducted before the negotiations. The entire correspondence and all negotiations were made available for our analysis. Additionally, we were allowed to take part in the hearings and document them with recordings so that they could be evaluated afterwards. The participants of the focus groups could be asked further questions to extract more in-depth insights and subjective evaluations and ascertain the need for the design patterns used.

To analyze our data and gain insight into the use of the design patterns, we conducted a structured qualitative content analysis according to Mayring (2014). The coding corresponded, on the one hand, to Yin (2018) and, on the other hand, was open for novel insights emerging from our data (Ozanne et al., 1992). The first author applied content analytical procedures to code and interpret the data in an iterative manner to identify the design pattern use. The goal of our coding procedure was to pinpoint the aspects that affect the design pattern value creation. During the coding, the first three authors were involved. The first author conducted the initial coding, which was verified by the coding of the co-authors to guarantee the analytical consistency of our results. If there were different codes, they were analyzed until a solution was found. Thus, the entire coding process followed an iterative cycle (Kazan et al., 2018).

### Findings of case analysis

**Fig. 3:**
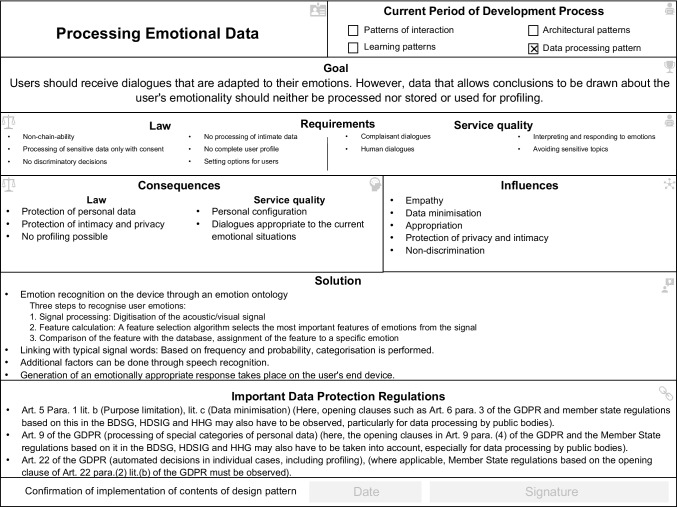
“Processing emotional data” design pattern

**Fig. 4:**
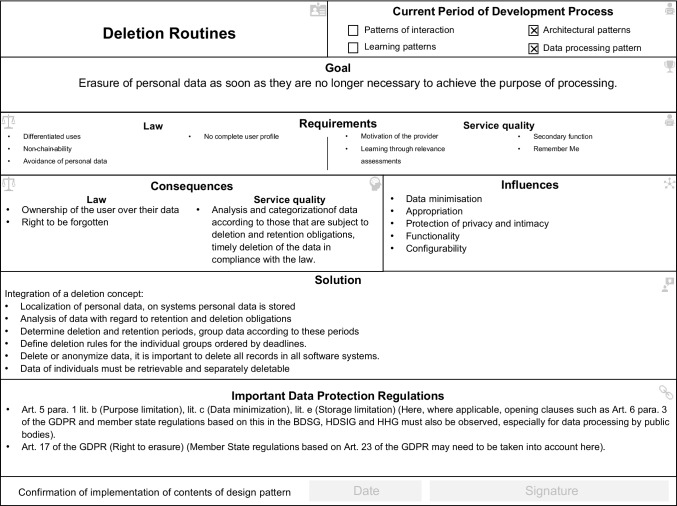
“Deletion routines” design pattern

Our case study provides comprehensive insights into the use of the legal design patterns. We have differentiated our findings into two phases which resulted from the data. First, in the development of the SPA in which developers have to follow legal specifications. Second, in the legal assessment of the developed SPA. We provided the developer and legal experts with a pattern repository, consisting of twelve legal design patterns. The design patterns were the result of an interdisciplinary research project between information systems, computer science and the law discipline (Dickhaut et al., 2020). During the project, the design patterns have already been proven to be useful (proof of value) for developing lawful technologies, and they are useful (proof of concept) in supporting developers in finding design solutions as shown through a rigorous experimental study (Dickhaut et al., 2020). The design pattern catalogue consists of the following design patterns: processing emotional data, privacy-friendly user profile, profiling on foreign devices, authorization management, deletion routines, integration of external payment data, data transfer to external devices, sensitivity to wake words, information assessment, private mode, individual assistance, and avoidance of personal data. Each design pattern consists of a unique name, seven content areas, and a signature field to confirm the design pattern implementation. To go into as much detail as possible about the use of the design patterns, in the following, we focus primarily on the use of the “processing emotional data” design pattern (see Fig. 3) and the design pattern “Deletion Routines” (see Fig. 4) as two representatives for the use of all twelve design patterns during the simulation study. Hence, we had to analyze both phases to understand how legal design patterns, as a whole instrument to develop lawful technologies, are used to support the development and assessment of novel technologies that enter the market.

#### Developing the smart personal assistant

In the development phase a learning assistant was developed for support during exam preparation in university teaching. The development of the SPA took four weeks (the elicitation of requirements took place in advance) and involved three developers working on it full time. The developers were provided with legal requirements, which were collected from teachers, students, and the legal department of the university. In addition to the requirements, we provided the developers with a legal design pattern catalogue that included proven solutions for recurring legal issues. The developers were free to use the design patterns during development. To analyze the case, we used the development documentation and conducted interviews with the developers to gain a comprehensive understanding of the development and the design pattern use.

In our case, all three developers struggled with implementing the legal requirements in the technology. To be able to do so, they first had to translate the law into machine-executable representations as programming code. For this, they first tried to understand and interpret the legal requirements. Particularly in situations with legal problems regarding the storage and processing of emotional data, where legal knowledge was required, the developers used the design pattern to point out proven solutions in understanding and interpreting the law. Data protection regulations, such as special protection of personal data, led to strict legal requirements for the SPA. In addition to the specifications for data storage, the data processing of emotional data was also decisive for its compliance with the university. In our case, the developers used the design pattern “processing emotional data” to find solutions that meet the strict legal requirements of the GDPR and the university (all of the following quotes have been translated into English):

"I have little knowledge of data protection information and often did not know how to implement the requirements in practice. The “processing emotional data” design pattern showed me approaches to solutions that I could use as orientation.” (Developer 2).

The application of the design pattern required the developers to apply the abstract legal requirements to concrete development solutions. In detail, the developer integrated emotion recognition on the device through an emotion ontology. This allows the system to adapt to the emotional state of the learner and, for example, recognize swear words and respond accordingly. Once a swear word is detected, the SPA does not record the next word frequencies, which means that no connection to the learner’s user profile is established. The design pattern solution suggests that the processing of emotional data is taken into account and gives approaches for its handling. As a result, the design patterns provided a direction for a solution but not a complete solution. Thus, we could observe a mismatch between understanding the law and its practice, which led to somewhat difficult decisions in the development for the developers due to a certain degree of perceived vagueness. More precisely, the legal requirements for the system were rather inaccessible to developers, making the transfer from law (legal requirement) to practice (implementation) challenging.

In the interviews, developer 2 and developer 3 compared the design patterns with the technical documentation, as documenting the steps taken throughout the development was a "mandatory obligation". In comparison to the documentation, the design patterns provided a benefit and thus an added value for the final development and achievement of a particular goal, whereas the documentation was very time consuming and did not necessarily add direct value to the system.

"Now we can simply use the design pattern as documentation, and we have killed two birds with one stone. On the one hand, we receive help in the development, and, on the other hand, we save ourselves the tiresome documentation". (Developer 3).

Thus, the design patterns were part of the documentation but extended the purpose of reusing the design knowledge into new contexts. However, the design patterns did not completely replace the technical documentation of the development, because they provide a solution scope to identify concrete solutions for the system to be developed.

In addition, the design patterns supported the developers in the reuse of the design knowledge through proven solutions. All developers used the design patterns frequently to get a range of possible solutions. The design patterns were always used if no solution was found for the problem to be solved. By using already proven solutions, the developers gained a feeling of security when approaching unfamiliar territory like legal requirements. To improve the storage and processing of this data, the development team used the “Deletion Routines” design pattern (see Fig. 4). In the case observation of the development, as well as in the interviews, we saw how the developers started to recognize the relevance and importance of legal requirements by using the design patterns.

"Finally, the legal requirements make sense, and I understand the purpose of their implementation." (Developer 2)

The design patterns guide the user to possible solutions and build specific domain knowledge. In summary, as shown by the following quote, design patterns indicate the reasons for the need to do something:

"I get further explanations and hints for each approach, so I can understand what the individual specifications in the pattern are necessary for." (Developer 1)

Hence, the design patterns show an approach that guides the user through implementing the legal requirements and integrating legal aspects into the technology.

#### Legal assessment

While the previous section highlighted the mechanisms of how design patterns facilitate the development of lawful IS, we now look at how design patterns are used for assessing the lawfulness of IS. To assess the lawfulness of the developed SPA, a simulation study was conducted, which is a well-established methodology for evaluating technologies among legal experts (Pordesch et al., 1999). The simulation study included a user study in which the system users, in our case students, used the SPA under real conditions. The use of the SPA served as a foundation to create simulated legal disputes. In these, the users contacted their lawyers regarding unnecessary data storage beyond the purpose it was actually intended for or discrimination in the application process because of bad performance during the SPA use. In our case, the assessment was a legal assessment of the previously developed SPA. This took place in four simulated court cases, which assessed the lawfulness of the learning assistant. In the simulation study, a court case is conducted under real conditions and the legality is negotiated. The participating lawyers and judges contribute their practical experience to the process and at all times behave the same as they would in a real court hearing. The actions of the four trials, unlike the other conditions, were invented and were based on trial experience. The first court case revolves around the disclosure of personal data beyond the use of the SPA. The second case negotiates whether the AI-based assistant may be used at all in the context of the teaching event. The third case analyzes the storage duration of personal data and whether this was observed. The fourth case discusses the right to delete the data and whether this is granted. Thus, as in the analysis of the development, we extracted major benefits of using the design patterns during the court cases, which we describe in detail in the following.

Like in a real court situation, the simulated court cases start with the lawyer’s statement of claim by presenting the lawyer’s view of the facts and the infringement. Thereupon, the written preliminary proceedings between the plaintiff’s lawyer, the defendant’s lawyer, and the judge start. The design patterns are first introduced in the written pre-negotiation. The defendant’s lawyer introduces the design pattern “Deletion Routines” (Fig. 4) in his or her ’statement of claim’ for his or her evidence. The design patterns are used as evidence for the practical implementation in the system and to demonstrate that no unnecessary data has been stored and processed beyond its intended purpose. Besides his or her written statement of defense, the lawyer attaches the design pattern. As soon as both parties have exchanged the first arguments and have presented their factual situation, the lawyer invites everyone to the oral hearing.

The use of design patterns, at the very least, documents that the developers took the legal aspects seriously during the system development and made an effort to adhere to previously considered lawful design knowledge. In the focus group discussion, the judge makes the following observation:

"The fact alone that the pattern has been taken into account in the development shows the importance of the protection of personal data." (Judge 2).

This leads to the fact that the discussion starts in favor of the developers. The use of the design pattern “Deletion Routine” already shows that the will was generally there to develop a lawful system. This can be used to the advantage of the defendant’s technology, especially at the beginning of a trial. When it comes to negotiating fines, there is often a question of whether the person in charge has even thought about the issue:

“Here you can explain the first step, which means that the fines will be reduced. The more specifically one can then explain this, the better the argumentation.” (Judge 1)

Lawyer 3 argues in response that the defendant who participated in the development can confirm that the design patterns were fully taken into account in the development. On page two of his or her statement of defense, he or she refers to the implementation of a deletion routine that provides "all data will be permanently deleted on a regular basis according to a predetermined deletion routine as provided for in the design pattern deletion routine". In court, the design pattern “Deletion Routine” is already introduced at the beginning by the judge (judge 1) in the reading of the action and the statement of the defendant. During the first 15 min of the trial, the subject returns to the design pattern "deletion routine" as evidence. With little time in the negotiation to react to arguments from the other side, the design pattern quickly provides the exact information that the defending lawyer needs at that moment. Thus, he or she argues the following: “The data that is not necessary will also be deleted. This is done automatically as can be seen in the design pattern […]" (minute 16:45 of the trial). Due to the clarity and the fact that all the patterns correspond to the same structure, the legal experts can quickly get the used information and build arguments. This link between law and technology leads to an understanding of how the system functions technically and provides an understanding of which legal requirements are observed and used for argumentation. In the focus group, lawyer 1 praises the clarity of the design patterns:

“[…] you can see that the instructions were followed to implement legal requirements and argue with it.” (Lawyer plaintiff 1)

In summary, in the oral hearings, arguments are presented on the basis of five design patterns. In the second oral hearing, four design patterns are used throughout the court proceedings for the argumentation. In both written procedures, patterns are also used to demonstrate the development of the lawful IT artifact. In addition to the statements of both parties, a neutral expert is asked to confirm the implementation of the design patterns. Although the design patterns are accepted as evidence, they do not confirm the actual implementation in the technology. On the one hand, the design patterns allow insights into the development; on the other hand, the patterns do not guarantee correct implementation, which is why the judge requires an expert’s statement on the implementation.

The design patterns are always used as soon as the technical details are negotiated and certain implementations are not clear, for example, to gain an understanding of the development process. In general, any court hearing aims to clarify the state of the facts so that both sides contribute their evidence and argumentation. The design patterns are used in this case to refer to the technical details within a lawyer’s argumentation. For this, the defendant uses the details of the design pattern, which provides the possibility to use expert knowledge in understandable language:

"Whenever I was at a loss with my arguments, I could find technical details of the programming in the design patterns and use them for my arguments." (Lawyer defendant 1)

The defendant lawyer uses patterns to form arguments, thus transferring complex technical insights of the design patterns into legal jargon. Hence, it is crucial that the content of the patterns can be understood and used by laymen to create a cognitive fit. Due to this, the patterns represent a possibility to impart knowledge and technical understanding (also for any general user from other domains).

"The technical information in the pattern is easy to understand […]." (Lawyer defendant 2)

"They offer background information about the development details." (Lawyer defendant 1)

In this way, a link to legal implementations in the technology can be drawn from the patterns in the case of technical points of attack.

“The patterns are, in the end, aid for finding the argumentation.” (Lawyer defendant 2)

“They offer information to write a statement of defense.” (Lawyer defendant 1)

Not only the lawyers are able to form arguments on the basis of the design patterns, but the judges are also able to use the design patterns to clarify the state of the facts. In one interview, judge 1 says:

"You can finally understand what purpose the data is needed for and no longer have the feeling that data storing is carried out without any purpose." (Judge 1).

The judge who tries to understand the implementation of the digital learning assistant can refer to the patterns in their questions to both parties. The questions primarily challenge whether the descriptions of the patterns were implemented in practice as described.

## Capturing the twofold value of design patterns through a theoretical model

**Fig. 5:**
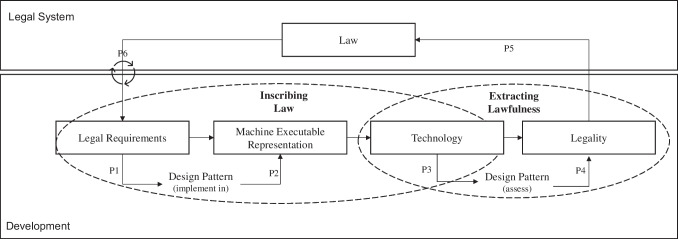
Theoretical model of legal design patterns for inscribing law and extracting lawfulness

Building upon the findings of our case study, we utilize the a-priori introduced cognitive fit theory to investigate how design patterns bridge the gap between the mental problem representation and the representation of the problem solvers, namely the developer and the legal experts. Legal experts and developers are expert in their own domain and have usually limited knowledge of the other discipline which leads to challenges of their mental representations. Hence, based upon our empirical findings and the grounding in cognitive fit, we develop a theory model that constitutes our central contribution of the present study (see Fig. 5). Bacharach (1989, p. 498) states in this context that ‘the primary goal of a theory is to answer the questions of how, when, and why, unlike the goal of description, which is to answer the question of what’.

First we explore why and when IS designers and lawyers utilize legal design patterns in design processes as well as legal assessment. Furthermore, we investigate underlying mechanisms on how design patterns affect IS designers and lawyers using cognitive fit theory, and it affects the overall the legal system. We describe this procedure as inscribing the law into the technology.

Second, whether something complies with the legal requirements is determined by legal experts. If there is a violation of the law, the definitive legality is only judged in court as part of a litigation process. We describe the second phase as extracting lawfulness from technologies, which describes the legal assessment of a technology. Scaffolded through the aforementioned cognitive fit theory, we built upon our findings following a theorical model, depicted in Fig. 5, which distills the key mechanisms of inscribing law into technology and extracting lawfulness from technology.

### Inscribing law into technologies

When mapping our observations with the cognitive fit theory, we can observe that developers have limited legal knowledge (internal representation) that makes it hard to transform their theoretical knowledge into practical design knowledge (Davern et al., 2012). Specifically, the scope for interpretation is too large to identify suitable design solutions. Through the design pattern use, the developers gain abstract design solutions, which they can adapt to their specific case (Taylor, 2001). This is important, since the law is typically technology agnostic and needs to be specified to both technology and use case. Hence, we propose our first proposition that design patterns extend the understanding of legal requirements through explicitly concretizing the law:

#### Proposition (P1)

Design patterns extend the developers’ internal representation through the specification of legal requirements.

One of the main challenges during development is the translation of real-world facts, which we define as the construct legal requirements into executable code, since development is a complex activity involving multiple stages and actors. We define the construct machine executable representation as the translation of machine language as a set of native instructions that carries out in hardware. Thus using the cognitive fit theory, our case reveals the development as a part of the problem-solving process by requiring the developer to find creative ideas and break them down into code fragments in as short a time as possible. Agarwal et al. (1996) developed a theoretical cognitive fit model that posits that superior problem-solving performance will happen when the problem-solving task and the problem-solving tool emphasize the same type of information. In our case, we observe the improvement of problem-solving through familiar presentation and examples of solutions in the design patterns. The patterns made proven design knowledge accessible to developers and created an opportunity to impart knowledge through proven solutions, resulting in our second proposition:

#### Proposition (P2)

Design patterns improve the developer’s problem-solving performance through the provision of proven solutions, i.e., designing a machine executable representation as code.

The relevance of improving the faithful inscription of legal requirements (P2) into code is also highlighted by the increasing (perceived) relevance of legal requirements. Typically, these requirements are perceived by developers as unavoidable and are only implemented towards the end of development due to a lack of time.

The design patterns helped by providing explanations and further information about the problem space and possible solutions as opposed to common logic methods, such as if/ then or flow charts. They offered a direction to follow and a way to think about the problem. The developer used existing knowledge, reflected upon it via the design pattern, and finally found a suitable solution for the problem at hand.

Coming back to answering the “how, why and when” questions by Bacharach (1989): The developers use the design patterns when they need to open the solution space to find ideas to solve the development problem. Following propositions P1 and P2 the design patterns are used to translate and understand the legal requirements into machine executable representation, thus serving as a support to translate the requirements into programming code. Using the cognitive fit theory, the design patterns extend the developers’ internal representation by making legal requirements specific (P1) and improve the problem-solving performance through providing possible solutions (P2).

### Extracting lawfulness from technologies

Our data shows that the design patterns often supported legal experts in the understanding of the technology. Mapping these insights with the cognitive fit theory, we observe that the design pattern act as a bridge between the external representation, the technology itself and the internal representation as the legal experts’ knowledge of the technological domain. Thus, the patterns support the formation of arguments during the trial because they contribute to a comprehensive mental representation of the technical domain, thus expanding the space of possible solutions. Based on the findings, we consider a cognitive fit between the understanding of a technology to be negotiated (mental representation) by legal experts and the clarification of the facts (problem-solving task) to be a decisive factor for a negotiation on the lawfulness of a technology in court. We define the negotiated artefact as the construct technology and the assessment as the construct legality. Thus, we propose our third proposition:

#### Proposition (P3)

Design patterns enable achieving a cognitive fit between understanding the technology (problem representation) and judging the lawfulness (problem-solving task).

The mental representation of assessing the technology’s legality consists of the internal representation of the problem domain, i.e., the existing knowledge about the technology to be negotiated, and the external representation of the problem, the technology itself (Shaft & Vessey, 2006). As soon as legal experts lack the necessary domain knowledge on complex socio-technical systems, the internal presentation of technical domain knowledge that can be accessed is limited. In our case, the external presentation of the technology consists of technical documentation and programming code, which is difficult to interpret and difficult to use for negotiation. Design patterns provide insights into the development procedure and, thus, white-box the development by acting as a bridge between internal representation and external representation and contribute to a better mental representation, which can be used for our task, namely the legal assessment. Hence, we propose our fourth proposition that further information in design patterns supports understanding the technology:

#### Proposition (P4)

Design patterns function as a bridge between internal and external representation and contribute to a better problem representation to decide on the lawfulness of the technology.

The interviews and evaluation of the court hearings show that the design patterns contribute to a comprehensive mental representation of the problem domain, thus expanding the space of suitable solutions and achieving a cognitive fit (Shaft & Vessey, 2006).

Coming back to the questions by Bacharach (1989), the main challenge during the legal assessment was to understand the technology and its underlying mechanisms (when). Thus, the legal experts use the design patterns to access the technologies’ function (why). As seen in P3 the patterns support the understanding of the technology (problem representation) and judging the lawfulness (problem-solving task). By acting as a bridge between the external and the internal representation the mental representation is improved (see Fig. 2). This is achieved by providing an understandable explanation on why the respective decisions were made during development. This knowledge allows the legal experts to argue based on design patterns.

### Bridging the gap between development and legal assessment

Combining the “inscription” and “extraction” themes discovered in our case, we constitute that design patterns serve as a bridge between technology and law for both developers and lawyers—either for developers to help ensure the technology is compliant with the law, which we describe as "inscribing law” into the technology, or for legal experts to assess the legality of these technologies.

As long as design knowledge exists in the thoughts of the developer, it resides on a rather nonmaterial level. The act of externalizing design knowledge transforms the tacit knowledge into something explicit by inscribing it into the technology, which, as can be expected, are design patterns. For developers, the design patterns, in practice, lead to the reuse of design knowledge. They provide a combination of action-oriented guidelines as well as explanations and effects for the developers to find a solution. The patterns may bridge the gap between initially unsolvable problems and the suitable design knowledge externalized in the design patterns.

By applying the patterns in a court case, the legal experts can use them to unfold underlying technical details, and the pattern provides additional information that can help understand and assess the system. Simply put, legal experts act upon the subject system and, together with the design patterns, try to figure out how the technical aspects of the system are related to the legal requirements. The reapplication reverses the process of unfolding the inscribed knowledge by using the design patterns during development and makes the knowledge accessible to the legal expert, which we call “extracting the lawfulness” of the developed technology. Legal experts come into contact with the system and are equipped with preconceived knowledge of social, technological, or legal knowledge. Hence, we propose:

#### Proposition (P5)

The assessment of IT legality shapes the interpretation of law and contributes to better representations.

Our analysis suggests that design patterns may act as a bridge between design knowledge and developed technology. In the development, the design patterns support the understanding and interpreting of the abstract legal requirements (P1) by providing proven design solutions to implement the legal requirements into machine-executable representations (P2). Hence, developers can inscribe the law into code, which forms the technology. Once developed, a novel technology, its legality, and practical use are not recognizable. This fact complicates the legal assessment of the technology. Legal experts, and oftentimes legal laymen, have to understand its overall functionality (P3) and find suitable arguments (P4). By applying the patterns in two different scenarios, we have seen that design patterns contain another characteristic. In our case, they can either act as support for the developers or as support for the assessment of the developed system (P5). Usually there is a time lag between the development of laws and legal requirements until a novel technology appears on the market. This can be seen, for example, with the GDPR, which appeared sometime – in year 2018—after data protection became relevant. The so-called “Brussels effect” underscores this aspect and even highlights that novel technologies comply with the GDPR irrespective if a service provider is subject to the GDPR (Peukert et al., 2022). Similarly, novel technologies have an impact on the development of further jurisdictions. For example, new types of technology may lead to the development of new guidelines and may concretize the existing legal situation. As seen in the upcoming relevance of voice assistants which lead the European Data Protection Board (2021) to provide guidelines for the lawful design of these systems. Hence, we propose our sixth proposition that law should be seen as an iterative cycle, which impacts the digital market as well as the development of these technologies and changes over time:

#### Proposition (P6)

Law and novel technologies influence each other in iterative processes.

## Discussion and contributions

### Discussion of findings

Design patterns provide a means to an end for accumulating design knowledge of IT artifacts in a way that is comprehensible enough to not only build IT artifacts but also communicate IT artifact design effectively across disciplines (Gamma, 1994). Accordingly, our study demonstrates how design patterns are a carrier of design knowledge, which serves as a mediator between developers and legal experts by acting as a bridge between the external representation (technology itself) and the internal representation (legal experts’ knowledge of the technological domain). Thus, on the one hand, design patterns provide guidance on the code and technical implementation. On the other hand, lawyers and judges are trained in technical understanding to negotiate the state of affairs in court.

#### Theoretical implications

We contribute with our study to theory by developing a theoretical model that considers the conceptual latitude (Burton-Jones et al., 2015) of the case through illustrating the interaction between novel technologies and existing legislation. For this, we extend cognitive fit theory to improve a missed cognitive fit (Shaft & Vessey, 2006) between internal and external representation by using design patterns as a bridge between two domains, namely law and technology. Our findings show that the use of design patterns in the context of lawful system development supports developers in understanding and implementing legal requirements in the technology (inscribing the law). At the same time, the same design patterns are useful during court cases to increase the understanding of technical mechanisms and thus facilitate the negotiation process and ultimately its judgement thereof. A connection between law and system development increases the cognitive fit of both parties. During development, the design patterns support the legal understanding of the developer and provide proven solutions for recurring legal problems. By providing additional legal design information, the developer can create a common understanding of the necessity of nonfunctional (primary legal) requirements that goes beyond the description of the goal state; the understanding of the context is fostered. The design patterns provide information related to the codependency of interdisciplinary details, such as possible consequences of the implementation for both disciplines.

During the legal assessment, the design patterns offered the benefit for the developers to show that they tried to address the legal requirements with a solution that had addressed at least a similar legal challenge elsewhere. Without considering each legal context, this is an indicator that can be used to argue for an intent to address the legal issues. This can be used in favor of the technology in court cases. The use of design patterns signaled to the legal experts that the legal requirements (problem space) and the tried and tested technical solutions (solution space) were at least attempted to be matched during the development (problem solution) (vom Brocke et al., 2020). This means that, within the assessment, the legal experts did not have to discuss whether legal requirements were considered and could rather focus on how they were instantiated. In other words, it was clear that the legal requirements were considered, and argumentation was formed based on technical details as evidence for the discussion in the court. Hong et al. (2004) argues that the same type of representation of the task to be solved and the mental representation is crucial for the cognitive fit. According to Hong et al. (2004), we assume that a bridge between the internal and external representation can support the cognitive fit because both representations should be on the same professional level of the domain knowledge. Formulating the interdisciplinary design patterns in a layman’s language has two major implications: On the developer’s side, they support developers in understanding complex legal requirements and adapting to the technology-neutral phrasings of laws and regulations. On the legal expert side, which includes prosecutors and judges, the design patterns support legal experts in understanding and building a mental representation of the technology (Vessey & Galletta, 1991) and supporting the negotiation process of the technology. For example, technical documentation of the code is usually poorly understood by lawyers and cannot be used to understand the problem domain, which would lead to no cognitive fit (Agarwal et al., 1996). Our study demonstrates that a design pattern is much more accessible and can be used to make arguments for and against design choices within the code.

Because the developers and the legal experts approach the materiality of systems with different goals in mind, they are faced with different possibilities for how to interact with design patterns and their respective system. Thereby, we observed that the kind of benefit and the purpose of design patterns depend on the context of the application and the user’s role. While developers used design patterns to develop a system and find solutions to problems, the exact same design patterns can offer other user groups, in our case legal experts, a completely different benefit. The legal experts used the design patterns to understand complex technologies in legal assessments. The design patterns lead to a more precise assessment and evaluation for the lawyers’ legal perspectives by making concrete implementations in the development recognizable and understandable. By using the design patterns to access the design knowledge of the development, the knowledge becomes perceptible (Hassan, 2016). The acquired knowledge can then be used for further work. The design patterns are abstract and can, therefore, be used in various application scenarios. Thereby one design pattern offers the explanation for many problems. Thus, design patterns can counteract one of the problems identified by vom Brocke et al. (2020) in the reuse of design knowledge and act as a bridge between what the software system is (development) and the understanding of its legal practice (assessment).

Summarizing, we lay the foundation with our theoretical model for empirical research but also work on practical research, since privacy aspects are and will remain an important part of the present and future. As such, we derive propositions building upon the developed theory propositions in our model (Bacharach, 1989). For instance, we use the second proposition to formulate the following hypothesis: Providing proven solutions through design patterns increases the development time. We expect that providing proven solutions will help developers find initial ideas and solve the problem based on them. This reduces the first step of understanding the problem. To test the design pattern effects during court cases, we suggest the hypothesis “The use of design patterns helps to formulate arguments faster in court”.

#### Implications on lawful system development and legal assessment

We contribute to practice by deriving insights into how design patterns support the development and legal assessment of technologies in court cases. The design patterns white-box the development of the technology by making the details of the development accessible to external parties, such as legal experts.

Due to the time lag of the appearance of novel technologies on the market, the law does not always provide usable support to develop legal requirements for what arises, among other things, from technology neutrality of the law. If we look at the specific case of SPAs, for example, there was, initially, great interest in their use. This was later destroyed by privacy scandals, and users became increasingly critical. Up until then, there were few legal guidelines that could be adhered to, and developers were often left to interpret and implement the legal requirements on their own. It was not until 2021 that the European Data Protection Board published Guidelines 02/2021 on Virtual Voice Assistants (European Data Protection Board, 2021), addressing the emergence of these new technologies. In comparison to other approaches, design patterns can be deployed much earlier and provide a bridge for precisely this time lag.

In addition to the possibility of integrating legal requirements in the development of new technologies at an early stage, design patterns stand out due to their special life cycle. The content development of design patterns is an agile process that can be integrated into development projects with little effort. Let us look at the design pattern development of Alexander (1977) in architecture and Gamma (1994) in systems development. Both see a major advantage of design patterns over other measures of codification and knowledge sharing in the fact that proven solutions to recurring problems are captured. Once a helpful template of a design pattern has been developed (which we will take up as future work later), developers can continuously collect and share proven (legal) design knowledge in design patterns and make it applicable for others. Petter et al. (2010) describe the life cycle of a design pattern as a continuous process consisting of the phases development, deployment, use, and evaluation. If there are any legal changes, for example, due to the revision of the GDPR or solutions in design patterns being improved by using the design pattern, they can be easily made, and the design pattern can be updated.

In our study, the lawful system development shows how the choice of using design patterns during the development phase can potentially have huge impacts in future legal disputes. These legal assessments are input for the overall legal system. They explain how the legal system follows the emergence of development processes and assessed system artifacts. By allowing us a greater conceptual latitude, we take on a more holistic perspective for both the development process and the overall legal system. Hence, we contribute to the discussion of linking system-theoretical insights (such as connection to the legal system) to process-theoretical outcomes for the lawful development of IT artifacts (Burton-Jones et al., 2015). Over time, a lack of legal knowledge and guidelines during the development of novel digital services lead to a trial-and-error phase, which result in experimental implementations of legal requirements. Naturally, some potential legal violations follow over time, as seen in the legal violations of Zoom in the European Union (Security Week, 2020). However, with the help of design patterns within lawful development, design knowledge can benefit from previously tried and legally assessed design patterns. With design patterns as a means to improve the cognitive fit between technical and legal knowledge, developers and legal experts can more easily assess each other’s work.

The simulation study and the design patterns used have been prepared and carried out in accordance with European law. Thus, the focus here is on the GDPR. The European legal system and the GDPR are some of the strictest legal systems in the world and their validity also has an impact on IT service providers and companies that offer their services to European users. In their study, Aljeraisy et al. (2021) compare the GDPR with various other legal systems, such as the California Consumer Privacy Act (CCPA) and Australian Privacy Principles (APPs), and show the transferability of the GDPR to the other systems. Thus, in comparison, the GDPR has a far wider scope.

#### Consequences of the use of design patterns during development and legal assessment

In the following, we will discuss the design pattern approach in comparison to other presented approaches and point out limitations of the approach. The advantage of the design pattern is the direct integration into the development. Developers can improve the development and integrate legal aspects into the system without additional effort. In addition, design patterns can implement law early, preventing costly penalties and changes to technology. Compared to requirement patterns, such as those used by Hoffmann et al. (2015), design patterns must rely on a satisfactory elicitation of legal requirements. However, the implementation of legal requirements strongly depends on the elicitation and formulation in an understandable form. With design patterns, only a small building block of the whole development process is considered. However, this focus also allows both developers and legal experts to benefit from the approach. Requirement patterns can involve legal experts in the creation of the pattern but in the end often offer little benefit for their practical work. Thus, the main limitation of the design pattern may lie in the limitation to actual development and in the fact that both developers and legal experts might blindly rely on the design pattern, which calls for research on how different stakeholders utilize the affordances offered by design patterns. In addition, when relating design patterns to the broader class of service design methods, we consciously limited the investigation to the isolated role of design patterns in development processes. However, we also call for research that integrates design patterns in existing frameworks (e.g., Teixeira et al., 2017) to better understand the impact of design patterns in a more holistic way.

In summary, design patterns are a common means of knowledge transfer in many disciplines, such as system development, architecture, and education. However, the use of design patterns also brings dangers with it. For example, design patterns do not embody a “timeless quality” (Sedig & Parsons, 2013) and have to be revised after a while. In particular, the legal content would have to be regularly reviewed and revised to guarantee the quality of the patterns. Without the date of creation, publisher, and information on the proof of the design pattern, it remains difficult to trust the design pattern blindly. Thus, participants in our simulation study were aware of the origin and authors of the design pattern in a way that would not necessarily be the case in practice.

### Limitations and future work

Our study has certain limitations that provide directions for future research. We provided insights into the holistic utilization of design patterns from the development to the legal assessment of a technology. In our study, the added value of design patterns was demonstrated in both cases. With the support of the design pattern, it was possible to develop a lawful technology that subsequently proved its worth in all four simulated court cases, and the design pattern also helped to support communication between developers and legal experts. However, it is important to note at this point that even if a system has been developed according to the best legal knowledge available, in practice, this does not automatically guarantee that the system will not have any violations of the law in the future. In practice, once someone suspects a violation of the law, the technology must be subjected to legal court cases and, during the negotiations and the legal discourse, a judge will decide upon the lawfulness of the system. The legal practice in negotiations argues the technology’s lawfulness based on the advocates’ knowledge to represent facts on behalf of their clients (Bellucci & Zeleznikow, 2005). Usually, lawyers apply their legal knowledge to the information that they receive from their clients by using documents such as contracts or documentation (Morcón et al., 2000). Design patterns and other comparable approaches will always be an interpretation of the law and the practical implementation remains a matter of interpretation in court.

In comparison to the EDPB guidance on the application of the GDPR to virtual voice assistants (European Data Protection Board, 2021), design patterns do not provide official guidance. The design patterns build on proven solutions and can thus be used much earlier than official guidelines. However, they cannot guarantee that they will not lead to infringements. Further research should look at the certification of existing design patterns and examine the issue in more detail because new technologies develop quickly, and the law should not lag behind this speed.

Finally, the design patterns in our case study were created specifically for lawful system development and are intended to assist developers in implementing lawful requirements. It is easy to observe phenomena and put them into a pattern-like form but much more difficult to use this knowledge to develop and explicate good design patterns (Sedig & Parsons, 2013). In the interviews and the focus group, the participants liked the clear presentation and layman’s language of the design patterns. These findings indicate the need for further work that takes a closer look at the presentation and requirements of helpful design patterns.

## Conclusion

In conclusion, this case study has shown how design patterns that include legal design knowledge may support bringing lawful technologies to the market. By developing a theoretical model that demonstrates the interaction between novel technologies and existing legislation, we demonstrate the strong interrelation between both disciplines. In terms of theoretical contribution, our lawful system development process provides a greater understanding of how design pattern artifacts influence future system development projects. In practice, the lawful system development shows how the choice of using design patterns during the development phase can potentially have huge impacts in future legal disputes. In addition to the added value of design patterns for the development of technologies, the simulation study has shown how design patterns can support legal experts in assessing the legality of complex technologies by making the development background transparent.

## Figures and Tables

**Fig. 6:**
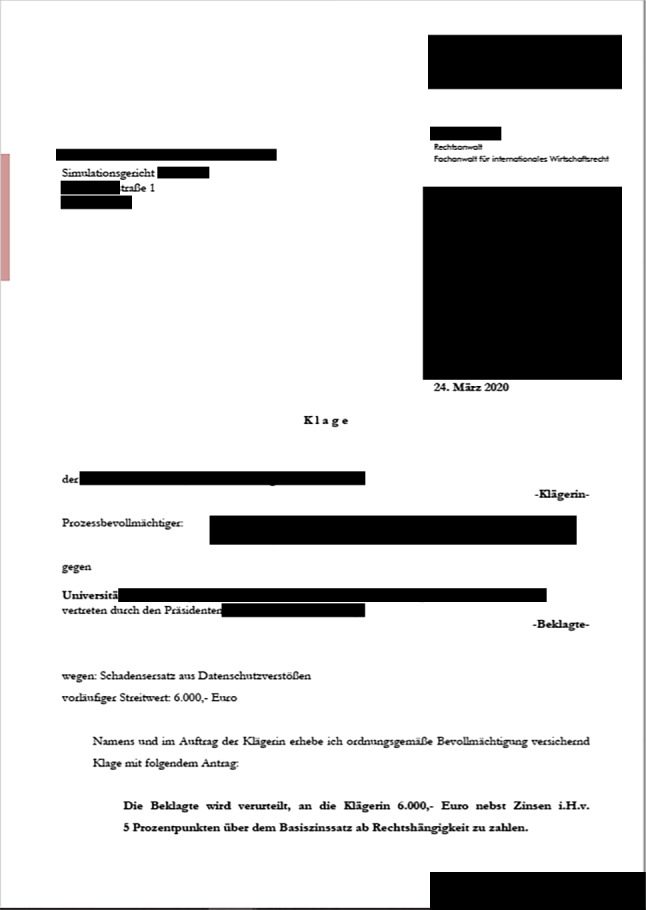
Statement of claim

**Fig. 7:**
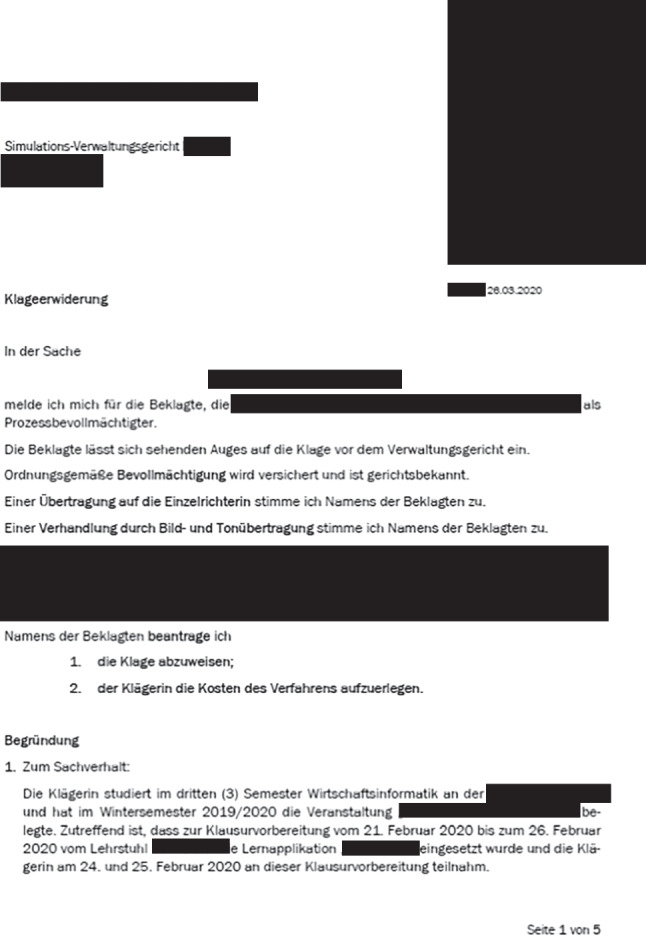
Statement of defense

**Fig. 8:**
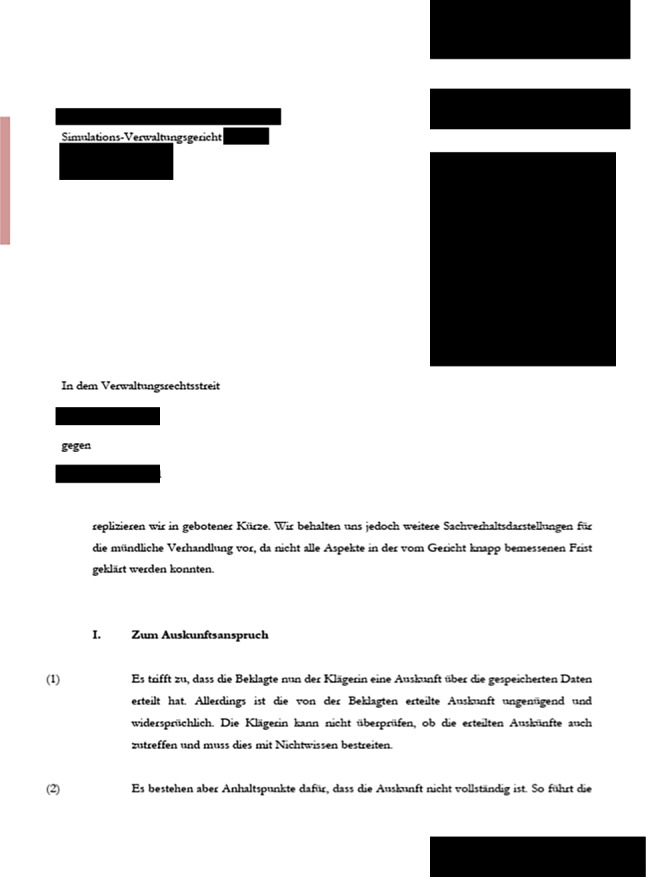
Replication

## Appendix 1

Table 3 presents an overview of fundamental constructs of our study and the developed theoretical model.

**Table 3:** Fundamental constructs of our study and theoretical model

Construct	Definition
Design pattern	Proven solutions to solve recurring problems and challenges; oftentimes represented as templates
Extracting lawfulness	Describes the legal assessment of a technology: whether something complies with the legal requirements is typically determined by legal experts who judge the technologies’ lawfulness
Legal design patterns	Proven IT solutions to solve recurring legal requirements and challenges while ensuring at the same time user requirements
External representation	The problem to be solved; including information such as explanations, documentations
Inscribing law	Development of lawful technologies that follow legal requirements. The procedure while developers decide to include legal aspects
Internal representation	Own understanding of the problem domain; depending on previous knowledge
Lawful	Legality of a system that meets the minimum legal requirements to be approved for the market
Machine executable representation	Machine language as a set of native instructions that carries out in hardware
Service quality	Key user expectations during the technology use related to usability and user experience

## Appendix 2

Court Documents.

Within the simulation study, we have received various documents. The documents reflect the realization of the simulation study as real court cases. The documents are written in German, as is the trial itself. The following documents are excerpts from a trial: statement of claim, statement of defense, and replication. We have anonymized the documents.

Figures 6, 7 and 8 presents court documents from our simulation study.

After the plaintiff has filed the lawsuit, the lawyer for the defense has the opportunity to submit the facts of the case from his point of view to the court and to introduce evidence here as well.

In his replication, the plaintiff’s lawyer comments on the statement of defense. This concludes the written preliminary proceedings, and the plaintiff is summoned to a court hearing.

## Footnotes

1 Researchers and IS practitioners often have a hard time with the language or terminology of lawful system design. To alleviate these aspects and to create a common understanding of the terms used in this study and later on developed theoretical model, we created Appendix 1, which contains a list of key constructs and their definitions that enables to check term definitions when they get lost or confused in terminology.

2 Nonetheless, we note that dialogical communication is typically a two-way process and confusion could also arise for lawyers through the technical jargon of (functional) requirements of a system that are typically specified by requirements engineers or system developers.
